# The Effect of Intravenous Magnesium Sulfate Infusion on Sensory Spinal Block and Postoperative Pain Score in Abdominal Hysterectomy

**DOI:** 10.1155/2014/236024

**Published:** 2014-03-19

**Authors:** Fatih Kahraman, Ahmet Eroglu

**Affiliations:** ^1^Private Guven Hospital, Anesthesiology, Trabzon 61000, Turkey; ^2^Karadeniz Technical University, Trabzon 61000, Turkey

## Abstract

*Aim*. The aim of this study was to investigate the effect of i.v. infusion of magnesium sulphate during spinal anesthesia on duration of spinal block and postoperative pain. *Methods*. Forty ASA physical status I and status II, aged between 18 and 65, female patients undergoing abdominal hysterectomy under spinal anesthesia were enrolled in this study. Patients in the magnesium group (Group M, *n* = 20) received magnesium sulphate 65 mg kg^−1^ infusion in 250 mL 5% dextrose at 3.5 mL/min rate, and control group (Group C, *n* = 20) received at the same volume of saline during operation in a double-blind randomized manner. Duration of sensory and motor block, systolic, diastolic, and mean arterial blood pressures, heart rates, pain scores (VAS values), and side effects were recorded for each patient. Blood and CSF samples were taken for analysis of magnesium concentrations. *Results*. Regression of sensorial block was longer in Group M when compared with that in Group C (175 ± 39 versus 136 ± 32 min) (*P* < 0.01). The VAS scores were lower in Group M than those in Group C at the 2 time points postoperatively (*P* < 0.01). *Conclusion*. 65 mg kg^−1^ of magnesium sulphate i.v. infusion under spinal anesthesia prolongs spinal sensorial block duration and decreases pain VAS scores without complication in patients undergoing abdominal hysterectomy.

## 1. Introduction 

Magnesium sulphate has been used as an adjuvant for perioperative analgesia [1–4]. Many clinical studies have demonstrated that i.v. magnesium infusion during general anesthesia reduced anesthetic requirement and postoperative analgesic consumption [1–9]. Relatively few studies have been investigated on the effects of magnesium sulphate infusion during spinal anesthesia [10–12]. A previous study demonstrated that i.v. magnesium sulphate infusion during spinal anesthesia improves postoperative analgesia in total hip replacement [12]. Apan and colleagues [10] reported that magnesium sulphate infusion started immediately after the spinal anesthesia and extended postoperatively for 24 h reduced analgesic consumption without any effect on spinal block in minor surgical procedures. Thus, the effect of magnesium infusion during spinal anesthesia on postoperative analgesia and duration of spinal block have not been fully determined yet. The aim of this study was to investigate the effect of i.v. infusion of magnesium sulphate during spinal anesthesia on postoperative pain and duration of spinal block in abdominal hysterectomy.

## 2. Materials and Methods

The study protocol was approved by the Institutional Ethics Committee and written informed consent was obtained from all patients. Forty ASA physical status I and status II, aged between 18 and 65 female patients undergoing abdominal hysterectomy under spinal anesthesia were enrolled in this study. Exclusion criteria included severe cardiovascular, renal and hepatic dysfunction, neuromuscular diseases, using calcium channel blockers, and inappropriate for spinal anesthesia.

After premedication with i.v. midazolam 0.03 mg kg^−1^, patients were transferred to the operating room. After placement of standard monitors included continuous electrocardiogram, pulse oximetry, and noninvasive blood pressure measurements, an 18-gauge IV cannula was placed. Spinal anesthesia was performed through L3-4 or L4-5 interspace in lateral decubitus position. Before the start of the fluid infusion 3 mL of blood sample was taken for analysis of preoperative serum magnesium concentrations, together with 2 mL CSF taken via the subarachnoid puncture. After dural puncture with a 25 G Quincke needle and 2 mL of clear CSF sample was withdrawn, 3 mL of hyperbaric bupivacaine 0.5% solution (Heavy Marcaine Spinal 0.5%, AstraZeneca AB) was injected into the subarachnoid space over 15 second. The lateral decubitus position was maintained to achieve the estimated level of the block; then patients were turned to supine before surgery started. The level of sensory block was evaluated by the loss of pinprick sensation (20-gauge hypodermic needle). Motor blockade was scored using a modified Bromage scale (0 = no motor block; 1 = inability to raise extended leg, able to bend knee; 2 = inability to bend knee, can flex ankle; and 3 = no movement). Readiness to surgery was defined as the presence of adequate motor block (Bromage score ≥2) and loss of pinprick sensation at T6. The inability to reach a sensory block at T6 within 30 minutes after spinal injection was considered to be a technical block failure and the patient was converted to general anesthesia.

Forty patients were randomly assigned to one of the two groups of 20 patients using sealed and numbered envelopes. Patients in the magnesium group (Group M, *n* = 20) received magnesium sulphate 65 mg kg^−1^ infusion in 250 mL 5% dextroz at 3.5 mL/min rate during operation. In control group (Group C, *n* = 20) 30 mL 0.9% sodium chloride in 250 mL 5% dextrose infusion was administered at 3.5 mL/min rate during operation in a double-blind randomized manner. Age, weight, height, ASA physical status, surgical time, duration of sensory and motor block, systolic, diastolic, and mean arterial blood pressures, heart rates, pain scores (VAS values), and side effects were recorded for each patient by an investigator blinded of the study groups. Hemodynamic variables were recorded at 10 times (baseline, and 5, 10, 15, 30, 45, 60, and 75 min after spinal anesthesia, at the end of the surgery, and at the PACU). Pain scores were evaluated using a 0–10 cm VAS (0 = no pain, 10 = worst pain imaginable) at the postoperative periods (at PACU, 2nd h, 4^th^ h, 8th h, and 12th h). When patients were transferred to PACU blood and CSF samples were taken again for analysis of magnesium concentrations.

Clinically relevant hypotension was defined as a decrease in systolic arterial blood pressure by 30% from baseline values, and it was initially treated with a rapid IV infusion of 200 mL lactated Ringer's solution; if this proven to be ineffective, an IV bolus of 5 mg ephedrine was given. Clinically relevant bradycardia was defined as heart rate decreases to less than 45 bpm, and it was treated with 0.5 mg IV atropine. Rescue analgesia with tramadol 50 mg i.v. was available when VAS scores were >3. Nausea and vomiting were treated with 4 mg ondansetron intravenously.

### 2.1. Statistical Analysis

Sample size was estimated using pain scores (VAS) as the primary variable. On the basis of a previous clinical experience and assuming an SD of 1 cm, 17 patients were required in each group to have a *β* error 0.2 to detect a difference of 1 cm on the VAS at the type 1 error 0.05 level of significance. The Kolmogorov-Smirnov test was used for normality of data distribution. Parametric data (age, weight, height, hemodynamics, VAS, and duration of sensory block) were compared using Student's* t*-tests between the two groups. Discrete variables (side effects) were compared using a chi-square test. Repeated-measures ANOVA was used to analyze hemodynamic variables over time between two groups. All data are presented as means ± (SD) or number. A *P* value ≤ 0.05 was considered as statistically significant.

## 3. Results

There were no significant differences between the two groups with respect to patient characteristic (age, weight, and height), ASA physical status, and surgery times (Table 1). The subarachnoid space could not be identified in one patient randomized to Group M, and one spinal block failed in group C. These two patients received general anesthesia, and they were excluded from the study. Thirty-eight female patients completed the study. All of the patients were operated for abdominal hysterectomy by the same surgeon.

**Figure 1:**
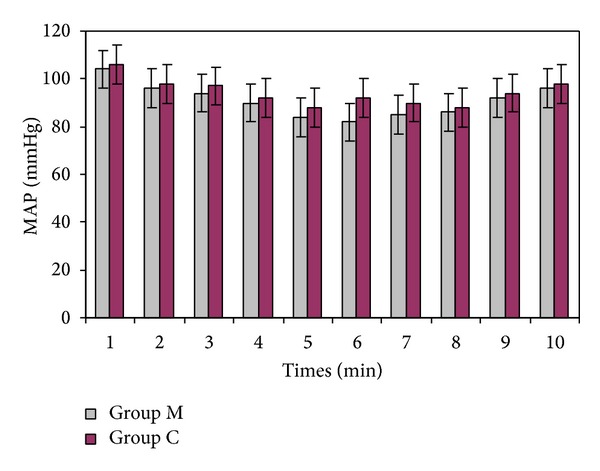
Mean arterial blood pressures (mmHg) of two groups, (1: baseline, 2: 5, 3: 10, 4: 15, 5: 30, 6: 45, 7: 60, and 8: 75 min after spinal anesthesia, 9: at the end of the surgery, 10: at the PACU).

**Figure 2:**
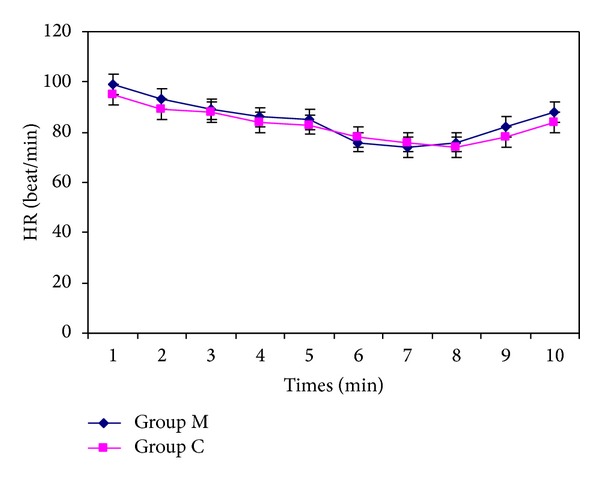
Heart rates (beat min^−1^of two groups, (1: baseline, 2: 5, 3: 10, 4: 15, 5: 30, 6: 45, 7: 60, and 8: 75 min after spinal anesthesia, 9: at the end of the surgery, and 10: at the PACU).

Three patients in Group M and two patients in Group C developed hypotension, and two patients in Group M and one patient in group C experienced bradycardia during surgery. In accordance with the study protocol, all events were treated. Mean arterial blood pressures and heart rates were similar in the two groups (Figures 1 and 2). There was only ones nausea and vomiting in two groups. There was not observed any other side effects.

The two groups were similar in terms of height of spinal block. The most significant difference was seen in duration of sensory block. Regression of sensorial block was longer in Group M (175 ± 39 min) when compared with that in Group C (136 ± 32 min) (*P* < 0.01). The VAS scores were lower in Group M than those in Group C at 2 h and 4 h after the surgery (*P* < 0.001). However, there was no difference at the other time points for VAS scores (Table 2).

Both plasma and cerebrospinal fluid (CSF) magnesium concentrations of two groups were shown in Table 3. The concentrations of magnesium both at baseline and at PACU were similar between the groups. There was no correlation between duration of sensorial block and magnesium concentrations in blood and CSF.

## 4. Discussion

This study showed that i.v. magnesium sulphate infusion during abdominal hysterectomy under spinal anesthesia prolonged the duration of sensory block and reduced postoperative pain scores without any notable side effects.

Magnesium sulphate has been used as an adjuvant for perioperative analgesia. Although the basic mechanism of analgesic effect of magnesium sulphate is unclear, it is presumed that its antagonist effect of NMDA receptors prevents the induction of central sensitization due to peripheral nociceptive stimulation and abolishes hypersensitivity. In many studies the effect of magnesium sulphate on postoperative analgesic consumption had been investigated under general anesthesia [1–9], but relatively few under spinal anesthesia [10–12]. In a previous study, Apan et al. [10] used intravenous magnesium sulphate after spinal anesthesia. In that study, immediately after spinal block patients received a 5 mg kg^−1^ bolus of magnesium sulphate followed by a 500 mg h^−1^ infusion or saline in the same volumes for 24 h, and postoperative analgesic consumption was significantly lower in the magnesium group. But, VAS scores in the two groups during the first 24 h after surgery were similar except at 12 h after surgery, and thus, it was assumed that the dosage of magnesium sulphate used was insufficient for postoperative analgesia. In our study we used total of 4550 mg magnesium sulphate for 70 kg during spinal anesthesia, in Apan et al. [10] study they used total of 1350 mg for 70 kg approximately. Magnesium dosage of their study was lower than that in our study. Adequate bolus and infusion doses of magnesium sulphate are important for effective analgesia. Hwang et al. [12] reported that 50 mg kg^−1^ magnesium sulphate for 15 min and then 15 mg kg^−1^ h^−1^ by continuous i.v. infusion until the end of surgery during spinal anesthesia reduced postoperative pain and analgesic consumption without complication. Seyhan et al. [7] compared the effects of three different dose regimes of magnesium sulphate on propofol requirements, hemodynamics variables, and postoperative pain relief in gynecological surgery. They reported that a single bolus injection at 40 mg kg^−1^ of magnesium sulphate reduced postoperative morphine consumption, and when this was followed by a maintenance infusion of 10 mg kg^−1 ^h^−1^, the effect was enhanced. Moreover, increasing the maintenance infusion to 20 mg kg^−1 ^h^−1^ provided no additional advantage and induced unwarranted hemodynamic effects.

In this study, patients in the magnesium group received magnesium sulphate 65 mg kg^−1^ infusion in 250 mL 5% dextrose at 3.5 mL/min rate during surgery under spinal anesthesia and in the control group patients received saline at the same volumes. We found that the duration of sensory spinal block with 65 mg kg^−1^ i.v. magnesium sulphate infusion was significantly longer than that in the group control (175 ± 39 min versus 136 ± 32 min, *P* < 0.01). To our knowledge, this is the first study reporting the prolongation of spinal sensory block duration with i.v. magnesium sulphate infusion under spinal anesthesia in abdominal hysterectomy. Hwang et al. [12] compared i.v. magnesium sulphate 50 mg kg^−1^ for 15 min and then 15 mg kg^−1 ^h^−1^ continuous infusion and saline under spinal anesthesia for total hip replacement. They reported that time to first pain sensation and spinal block height was similar in the two groups. In a previous study Apan et al. [10] reported that there was no significant difference between the magnesium sulphate and the control group in terms of spinal block height or the duration of motor block. The different outcome of our study may be explained by the dosage of magnesium sulphate.

In this study we measured magnesium concentrations both in blood and in cerebrospinal fluid (CSF). Patients in the Group M had a bit higher blood magnesium concentration than those in Group control, but there was no side effect associated with hypermagnesemia. Magnesium causes a dose-dependent negative inotropic effect, and a peripheral vasodilatory effect [13, 14]. Theoretically, minor side effect of parenteral magnesium such as flushing, nausea, and headache are expected at the serum magnesium level above 2 mmol L^−1^, and potentially life-threatening complications, primarily involved in the cardiovascular and neuromuscular system, occur when serum magnesium concentrations exceed 5 mmol L^−1^ [15]. In this study, the magnesium concentration in the Group M was 1.7 ± 0.6 mmol L^−1^, which is below the level of minor side effects. In our study, arterial blood pressure and heart rates were similar in the magnesium and control groups. On the other hand, there are few studies that analyzed CSF characteristics in patients and that have investigated the effect of magnesium levels in CSF on the spinal block. In some studies a direct correlation had been observed between the CSF density and the number of dermatomes blocked under spinal anesthesia [16, 17]. Echevarria et al. [18] investigated the influence of CSF composition on sensory and motor block in patients with diabetes mellitus under spinal anesthesia. The level of potassium, sodium, magnesium, and calcium in CSF was considered markers of possible changes in nervous conduction. They reported that there was no difference in the CSF levels of these elements. In our study, there was no correlation between the duration of sensorial block and magnesium concentrations in blood and CSF.

In conclusion, 65 mg kg^−1^ of magnesium sulphate i.v. infusion under spinal anesthesia prolongs spinal sensorial block duration and decreases pain VAS scores without complication in patients undergoing abdominal hysterectomy. This outcome can investigate with different dosage of magnesium sulphate in further studies.

## Figures and Tables

**Table 1:** Demographic and surgical characteristics of two groups.

	Group M (*n* = 19)	Group C (*n* = 19)	*P* value
Age (years)	43 ± 5	41 ± 6	NS
Weight (kg)	70 ± 8	72 ± 9	NS
Height (cm)	162 ± 9	165 ± 7	NS
ASA (I-II)	10/9	11/8	NS
Duration of surgery (min)	76 ± 28	72 ± 23	NS

NS: not significant.

**Table 2:** Regression of the spinal block and pain scores (VAS) of two groups.

	Group M (*n* = 19)	Group C (*n* = 19)	*P* value
Duration of sensorial block (min)	175 ± 39	136 ± 32	*P* < 0.01
Pain scores (VAS) (0–10) at PACU	0 ± 0	0 ± 0	NS
At 2 h	2 ± 1	6 ± 2	*P* < 0.001
At 4 h	2 ± 1	5 ± 2	*P* < 0.001
At 8 h	1 ± 1	1 ± 1	NS
At 12 h	1 ± 1	1 ± 1	NS

NS: not significant.

**Table 3:** Magnesium concentration of two groups.

	Group M	Group C	*P* value
Baseline plasma Mg (mmol L^−1^)	1.6 ± 0.5	1.7 ± 0.3	NS
Baseline CSF Mg (mmol L^−1^)	2.6 ± 0.3	2.5 ± 0.4	NS
PACU plasma Mg	1.7 ± 0.6	1.6 ± 0.4	NS
PACU CSF Mg	2.7 ± 0.4	2.5 ± 0.5	NS

NS: not significant.
